# Evaluating delay of gamma oscillations in patients with schizophrenia using evoked response audiometry system

**DOI:** 10.1038/s41598-022-15311-6

**Published:** 2022-07-05

**Authors:** Masaya Yanagi, Aki Tsuchiya, Fumiharu Hosomi, Toru Terada, Satoshi Ozaki, Osamu Shirakawa, Mamoru Hashimoto

**Affiliations:** 1 Department of Neuropsychiatry, Faculty of Medicine, Kindai University, 377-2 Ohnohigashi, Osaka-sayama, Osaka 589-8511 Japan; 2 Izumigaoka Hospital, Izumi, Osaka Japan

**Keywords:** Schizophrenia, Biomarkers, Neurophysiology

## Abstract

Impaired gamma oscillations found in a 40-Hz auditory steady-state response (ASSR) in patients with schizophrenia are the robust findings that can be used for future biomarker-based therapeutics. To apply these significant observations into the clinical practice, a clinical system for evoked response audiometry (ERA) may be available. In this study, the delayed 40-Hz ASSR, which was reported as a potent biomarker for schizophrenia, was examined using the ERA system in patients with schizophrenia and its clinical relevance was investigated. The phase of ASSR was significantly delayed in patients with schizophrenia compared with the healthy subjects. The delayed phase was associated with severity of the disease symptoms in the patients. A phase delay with aging was found in healthy subjects, but not in patients with schizophrenia. These findings show availability of the ERA system to identify the delayed 40-Hz ASSR and its clinical implication in patients with schizophrenia. Further applications of the ERA system in clinical psychiatry are warranted in developing biological assessments of schizophrenia with 40-Hz ASSR.

## Introduction

Impaired gamma oscillations are a promising biomarker^1–6^ that can be used for future stratified medicine on the basis of biological assessments of patients with schizophrenia^7–10^. Gamma oscillations are the neural rhythmic activities with gamma frequency range (30–200 Hz), which play a role in information processing in higher-order brain functions such as perception, attention, and memory^11^. Auditory steady-state response (ASSR), a neural synchronization to periodic tone stimuli, can probe the ability to generate gamma oscillations using the stimuli modulated at the gamma frequencies. The impaired gamma oscillations detectable by 40-Hz ASSR in patients with schizophrenia are the robust findings^12^ that are reportedly associated with the core clinical features of schizophrenia^13–19^. Therefore, the impaired gamma oscillations captured by the 40-Hz ASSR may indicate underlying abnormalities in brain functions associated with the clinical manifestations of schizophrenia.

Since an initial study with electroencephalography (EEG) reported the reduced and delayed synchronization of 40-Hz ASSR in patients with schizophrenia^20^, the impairments of 40-Hz ASSR have been largely reported as reductions of phase-locking factor (PLF) and evoked power in patients with schizophrenia^12^. The PLF is defined as the cross-trial phase consistency of EEG activity corresponding to the modulation frequency rate of the tone stimuli, while the evoked power refers to stimulus-related changes in EEG power that are phase-locked to the tone stimuli^21^. In addition to these potent biomarkers, a recent study reported another biomarker—phase-locking angle (PLA), which is defined as the difference in the phase angle of ASSR between a subject and the reference group^22^. It reported that the PLA of 40-Hz ASSR was delayed in patients with schizophrenia compared to the healthy controls. Moreover, the delayed PLA had the greater sensitivity as a biomarker to schizophrenia than the reductions of the PLF and evoked power in the 40-Hz ASSR^22^. Nonetheless, the clinical implication for the delayed 40-Hz ASSR remains to be elucidated in terms of how it may help assess patients with schizophrenia. To investigate the clinical usability of the delayed 40-Hz ASSR in this complex disease, a configuration that can briefly assess the 40-Hz ASSR will be valid to study at clinical practice.

The ASSR potentials are largest in humans when the periodic auditory stimuli are presented at the frequency of around 40 Hz^23^. This characteristic of 40-Hz ASSR has been plausibly used for evoked response audiometry (ERA) in clinical otolaryngology. The medically approved device for ERA has a modified set of 40-Hz ASSR to test hearing loss at awake condition by judging the presence or absence of the phase-locked response. However, this ear test works under the condition that the subject has a proper cerebral function to transmit the auditory stimuli to the EEG signals. In the case of a standard device for ERA, Audera, the modified set for 40-Hz ASSR consists of tone stimuli with 100% amplitude and 10% frequency modulation rate at 46 Hz. In our previous application study with the ERA device, several patients with schizophrenia did not show the phase-locked response due to aberrant gamma oscillations in the ASSR elicited by a basic 40-Hz tone and the modified 46-Hz tone preset in the device^24^. This finding opens up the availability of ERA system to identify the impairments of ASSR with approximately 40 Hz stimulation in patients with schizophrenia. While analyzing the absence/presence of phase-locked response in the ASSR, the ERA system calculates the phase of ASSR, which represents the time-lag between the periodic tone stimuli and the neural response at the modulation frequency. If this phase provided by the ERA system is available to detect the delayed 40-Hz ASSR in patients with schizophrenia, the utility of ERA system that has globally established in clinical otolaryngology could be further developed for the psychiatric disease. Therefore, we applied the ERA system for patients with schizophrenia to capture the delayed ASSR using the basic 40-Hz and modified 46-Hz tone stimuli, and examined the clinical relevance with the delay.

## Results

### Delayed ASSR in patients with schizophrenia

**Figure 1:**
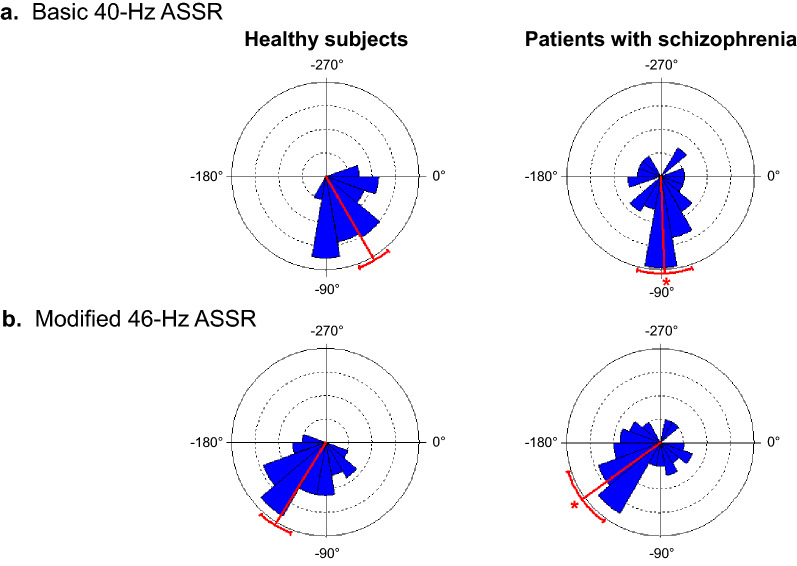
Comparison of phases between patients with schizophrenia and case-matched healthy subjects in the basic 40-Hz ASSR (**a**) and the modified 46-Hz ASSR (**b**). The rose diagrams depict the distributions of the phase of ASSR in healthy subjects (left panels) and patients with schizophrenia (right panels). Each area of wedges in the diagrams represents the number of subjects who fall into each bin of the phases. The phases were significantly delayed in patients with schizophrenia compared with healthy subjects in both ASSRs. The red bar line and arc represent the mean of the phases of ASSR and the 95% confidence limit, respectively. *p < 0.05.

The phase was significantly delayed in the patients with schizophrenia (− 87.9° ± 52.7) compared with the case-matched healthy subjects (− 60.0 ± 31.5) in the basic 40-Hz ASSR (*F*_1,78_ = 8.2, *p* = 0.006) (Fig. 1a). The significant phase delay of the patients was also observed in the modified 46-Hz ASSR (patients with schizophrenia − 144.3 ± 57.1, healthy subjects − 121.6 ± 33.9, *F*_1,78_ = 4.6, *p* = 0.04) (Fig. 1b).

### Delayed ASSR with disease severity in patients with schizophrenia

**Figure 2:**
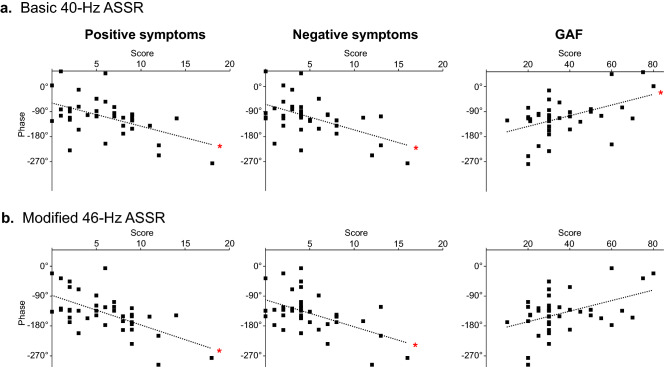
Phase delay with severity of the disease in patients with schizophrenia. The delayed phase was significantly correlated with positive and negative symptoms in patients with schizophrenia both in the basic 40-Hz ASSR (left and middle panels of **a**) and in the modified 46-Hz ASSR (left and middle panels of **b**). The delayed phase was also significantly correlated with lower GAF scores of patients in the basic 40-Hz ASSR (right panel of **a**). *p < 0.005.

To characterize the clinical relevance with the delayed ASSR, the correlations were examined between the phase of ASSR and clinical variables. A significant correlation emerged between the phase and the BPRS total scores in the basic 40-Hz ASSR (*r* = 0.42, *p* = 0.001) and modified 46-Hz ASSR (*r* = 0.50, *p* = 0.00009) in the patients with schizophrenia. A dimensional model for the BPRS item scores showed that the phase was significantly correlated in both ASSRs with those for Thinking Disturbance, i.e., positive symptoms [40-Hz ASSR (*r* = 0.45, *p* = 0.0005) and 46-Hz ASSR (*r* = 0.58, *p* = 0.000003)] and for Withdrawal-Retardation, i.e., negative symptoms [40-Hz ASSR (*r* = 0.47, *p* = 0.0002) and 46-Hz ASSR (*r* = 0.52, *p* = 0.00004)] (Fig. 2a,b, left and middle panels), but not for the other two dimensions: Anxious Depression [40-Hz ASSR (*r* = 0.15, *p* = 0.42) and 46-Hz ASSR (*r* = 0.10, *p* = 0.71)] and Hostile-Suspiciousness [40-Hz ASSR (*r* = 0.25, *p* = 0.10) and 46-Hz ASSR (*r* = 0.26, *p* = 0.09)]. The GAF score was significantly associated with the phase in the basic 40-Hz ASSR (*r* = 0.46, *p* = 0.0003) (Fig. 2a, right panel). This association of phase with the GAF score was nominally significant in the modified 46-Hz ASSR (*r* = 0.31, *p* = 0.03), although the significance has disappeared after Bonferroni correction (Fig. 2b, right panel).

### Other clinical variables and the phase of ASSR

Although nominal significances were found in the correlation between the phase and the chlorpromazine equivalent dose in the patients in both ASSRs [40-Hz ASSR (*r* = 0.31, *p* = 0.03), 46-Hz ASSR (*r* = 0.30, *p* = 0.04)], they disappeared after Bonferroni correction. A nominally significant correlation was noted between the phase and the illness duration in the basic 40-Hz ASSR (*r* = 0.29, *p* = 0.05), however it disappeared after Bonferroni correction. There was no significant correlation between the phase and the illness duration in the modified 46-Hz ASSR (*r* = 0.23, *p* = 0.14).

### Delayed ASSR with aging in healthy subjects

**Figure 3:**
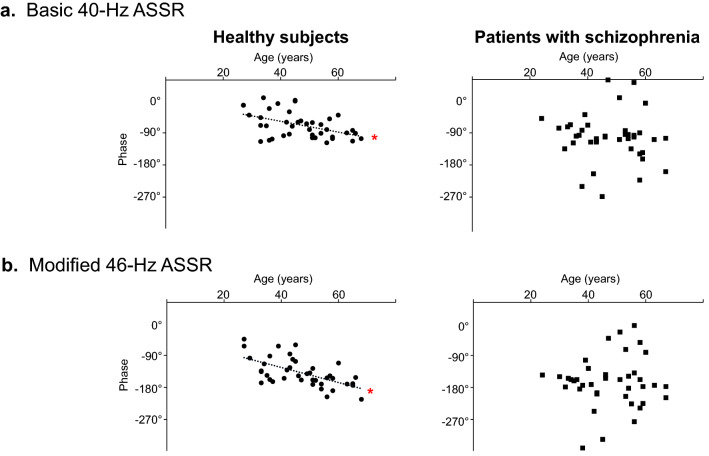
Phase delay with aging. A delayed phase with aging was significant in healthy subjects both in the basic 40-Hz ASSR and in the modified 46-Hz ASSR (left panels of **a** and **b**). This correlation was not observed in either of the ASSRs in patients with schizophrenia (right panels of **a** and **b**). *p < 0.005.

In the healthy subjects, the phase was significantly correlated with age in the basic 40-Hz ASSR (*r* = 0.49, *p* = 0.0001) and modified 46-Hz ASSR (*r* = 0.61, *p* = 0.0000005) (Fig. 3a,b, left panels). In contrast, there was no significant phase correlation with age in the patients with schizophrenia in either 40-Hz ASSR (*r* = 0.25, *p* = 0.10) or 46-Hz ASSR (*r* = 0.19, *p* = 0.27) (Fig. 3a,b, right panels). Nominal significance was noted for the phase difference between the sex in the healthy subjects in the basic 40-Hz ASSR (male: − 68.0 ± 23.9, female: − 47.2 ± 36.0, *F*_1,38_ = 4.3, p = 0.04); however, it disappeared after Bonferroni correction. No significant phase difference was found between the sex in the healthy subjects in the modified 46-Hz ASSR (male: − 140.9 ± 38.8, female: − 119.2 ± 34.0, *F*_1,38_ = 3.2, p = 0.08). There were no significant phase differences between the sex in both ASSRs in the patients with schizophrenia (40-Hz ASSR [male: − 88.5 ± 33.9, female: − 88.9 ± 53.1, *F*_1,38_ = 0.0, p = 0.98], 46-Hz ASSR [male: − 157.4 ± 39.9, female: − 154.7 ± 55.8, *F*_1,38_ = 0.0, p = 0.89]).

## Discussion

This is the first study that demonstrates the availability of ERA system for evaluating the delayed 40-Hz ASSR in patients with schizophrenia. Results showed that the delayed ASSR in patients with schizophrenia support the previous findings of expertized EEG studies. Moreover, the clinical significance of the delayed 40-Hz ASSR was examined in this study.

### Comparison with previous ASSR studies

Kwon et al. initially discovered the delayed phase of 40-Hz ASSR in patients with schizophrenia^20^. They used intermittent tone stimuli for the 40-Hz ASSR and found that patients with schizophrenia had delayed onset of phase synchronization and delayed desynchronization of ASSR^20^. Subsequently, Roach et al. developed this finding using a new parameter, PLA^22^. They showed that the PLA of 40-Hz ASSR was delayed in patients with schizophrenia^22^ and that the delayed PLA had the greater potential utility when compared with the reductions in PLF and evoked power, as the disease biomarker for schizophrenia^22^. Our results using the ERA device reproduced the delayed phase of patients with schizophrenia in the basic 40-Hz ASSR and also in the modified 46-Hz ASSR. According to the previous findings in the ASSR of patients with schizophrenia, the impairments of PLF and evoked power are not restricted to 40-Hz, but to the distributed frequency range around 40-Hz^25–27^. Taken together, it is suggested that the impaired ASSR including the phase delay exists in the slower gamma frequency range around 40-Hz in patients with schizophrenia. In this case, the consistency of our results with two measures of ASSR around 40-Hz may be explained by the excellent test–retest reliability that was reported on the PLA of 40-Hz ASSR both in healthy subjects and patients with schizophrenia^28^. Such an excellent reproducibility in measuring the phase of ASSR will support the utility of phase delay as a biomarker for schizophrenia as shown in the previous report^22^.

### Clinical implication of the delayed 40-Hz ASSR

A clinical relevance of the delayed 40-/46-Hz ASSR was shown in this study. The association of delayed phase with the positive and negative symptoms was detected in both ASSRs in the patients with schizophrenia. The concurrent associations with these two representative aspects of schizophrenia suggest the disease features of patients who had poor clinical outcomes involving severe psychosocial withdrawals and sustained delusion/hallucination. Although cognitive dysfunction was not assessed in these patients, the delayed phase was associated with lower GAF scores in the basic 40-Hz ASSR. Conversely, the association between phase and GAF scores was not significant in the modified 46-Hz ASSR, which may suggest that the basic 40-Hz ASSR is more suitable than the modified 46-Hz ASSR for comprehensively assessing the pathophysiology of patients with schizophrenia. Previous ASSR studies have reported that the impaired gamma oscillations indexed by the PLF and evoked power were associated with poor social functioning^15,19^ as well as positive^14,17–19^ and negative^13^ symptoms and cognitive dysfunction^15,16^ in patients with schizophrenia. These lines of evidence suggest that the impaired, namely, reduced and delayed, gamma oscillations were accompanied by severe psychosocial dysfunctions in patients with schizophrenia.

In the healthy subjects, the phase was delayed with aging both in the basic 40-Hz and modified 46-Hz ASSRs. A previous study reported that the PLF and evoked power were reduced with aging in 40-Hz ASSR in healthy subjects^29^. Combined with our results, it is suggested that the gamma oscillations deteriorate with aging in healthy populations as shown by the reduced and delayed 40-Hz ASSR. In contrast, our results in patients with schizophrenia did not show the phase delay with age in either 40-Hz or 46-Hz ASSR. This could have been because the impaired gamma oscillations already progressed at a younger age when the patients were clinically manifesting psychosocial dysfunctions. Considering the clinical implications of the impaired gamma oscillations in schizophrenia and the deterioration of gamma oscillations with aging in healthy populations, impaired gamma oscillations may be pathophysiologically involved in the classical disease concept of schizophrenia, which was once called dementia praecox whose psychosocial functioning declines early in life. The delayed gamma oscillations captured by the 40-Hz ASSR may be used in the biological assessment of the psychosocial dysfunctions of patients with schizophrenia and their improvement.

### Possible application of ERA system

The ERA system has the potential to serve for future biomarker-driven therapeutics in clinical psychiatry. This system has an advantage of being widely used in clinical otolaryngology, which makes it easy to apply for clinical practice in psychiatry. The global availability of this system at many hospital facilities makes it a potent platform for wide-reaching clinical trials on patients with schizophrenia and for expaning these trials into daily clinical practice. Although available evidence in patients with schizophrenia has not suggested a major impact of current antipsychotics on the 40-Hz ASSR^11^, 40-Hz ASSR could be modulated by GABA and glutamate neurotransmissions^10,30–32^. The delayed gamma oscillations detectable by the ERA system may be developed as a biomarker for tuning the impairments of these neurotransmissions in patients with schizophrenia. In addition, the automated analysis system adopted in the ERA device is suitable for briefly assessing the gamma oscillations in clinical settings. The impaired gamma oscillations detectable in the device as the absence of phase-locked response and phase delay can be easily visualized for the patient and attending psychiatrist as the impairments to target for treatment. Moreover, the brief assessment of gamma oscillations using the ERA system could be beneficial for the practical use where immediate therapeutic interventions are required for patients. The ERA system whose utility has been clinically established in otolaryngology has the potential to facilitate the clinical applications of gamma oscillations toward biomarker-driven therapeutics on the psychiatric disease.

### Limitations

This study has a few limitations. Firstly, the analytical method of the ERA system differs from that of the previous ASSR studies for schizophrenia. While the previous studies have adopted a time–frequency decomposition analysis using the discrete tone stimuli^21^, the ERA system provides only single summary results for the continuous tone stimuli by the limited analysis program implemented in the device. Due to this methodological difference, the phases of ASSR obtained by these two methods could be different. Secondly, all of the patients with schizophrenia were administered with antipsychotics in this study. Therefore, we cannot exclude the confounding effects of antipsychotics on the delayed ASSRs. Third, this is a cross sectional study that focused on chronic patients with schizophrenia. We recruited the patients whose symptoms were relatively fixed to address unstable course of the disease symptoms of schizophrenia. Longitudinal studies such as follow-up of patients with the first episode schizophrenia will be needed to examine the recoverability of delayed 40-Hz ASSR accompanied by improving the disease symptoms.

## Conclusion

This study revealed the availability of ERA system to assess the delayed 40-/46-Hz ASSR for patients with schizophrenia. The ERA system available in the current clinical settings may be useful in determining how the gamma oscillations help in the clinical assessment for the treatment of patients with schizophrenia. Further clinical applications of 40-/46-Hz ASSR with the ERA system will be warranted for developing biomarker-driven therapeutics in clinical psychiatry.

## Methods

### Subjects

Forty patients with schizophrenia (21 men and 19 women) aged between 24 and 67 years (mean ± SD, 47.5 ± 10.8) and 40 case-matched healthy subjects (21 men and 19 women) aged between 27 and 68 (47.0 ± 11.4) years were recruited in this study. Clinical information for each patient was obtained from the clinical psychiatrist in charge based on the detailed clinical observations during hospitalization and/or long-term follow-up appointments during outpatient treatment. Each patient was diagnosed on the basis of the DSM-5 criteria^33^ with the verification performed by two experienced research psychiatrists. The clinical symptoms and social functioning were assessed using the Brief Psychiatric Rating Scale (BPRS)^34^ and the Global Assessment of Functioning (GAF), respectively. The BPRS item scores were classified into a four-dimensional model^35^; Thinking Disturbance (hallucinatory behavior, unusual thought content, and conceptual disorganization), Withdrawal-Retardation (emotional withdrawal, blunted affect, and motor retardation), Hostile-Suspiciousness (hostility, suspiciousness, and uncooperativeness), and Anxious Depression (anxiety, guilt feelings, and depressive mood). Thinking Disturbance and Withdrawal-Retardation correspond to positive and negative symptoms, respectively^36^. The summary of these clinical variables are described in Table 1. All patients were administered with antipsychotics and were in a stable phase of the disease. None of the participants in this study had history of auditory disorders, neurological disorders, electroconvulsive therapy, or substance/alcohol abuse. The healthy subjects had no history of psychiatric, neurological, or auditory disorders. A complete description of the study was provided to each participant, and a written informed consent was obtained from each participant. This study was approved by the Ethics Committee of the Kindai University Faculty of Medicine and conducted in accordance with the ethical principles of Declaration of Helsinki and its subsequent amendments.

**Table 1:** Clinical variables in patients with schizophrenia and case-matched healthy subjects.

	Patients with schizophrenia		Healthy subjects	
	Mean	SD	Mean	SD
Age, years	47.5	10.8	47.0	11.4
Illness duration, years	23.8	11.6		
BPRS, total scores	22.5	11.6		
**BPRS, four-dimensional model**				
Thinking disturbance^a^	6.0	4.1		
Withdrawal-retardation^b^	5.0	3.9		
Hostile-suspiciousness	2.7	2.9		
Anxious depression	2.9	2.2		
GAF score	36.2	16.6		
Antipsychotics^c^, mg/day	815	572		
Sex	21 men, 19 women		21 men, 19 women	

^a^Corresponding to positive symptoms.

^b^Corresponding to negative symptoms.

^c^Chlorpromazine equivalent dose.

### ASSR measurements

ASSR measurements were performed using Audera (Grason-Stadler Inc., MN), the standard device for ERA, in accordance with the custom protocol of this device with a minor modification. Details of the ASSR measurements are described in our previous study^24^. Briefly, the ASSR was performed with an instruction to sit and relax on a chair, keep their eyes closed, and remain motionless to avoid muscular artifact generation. The auditory stimuli were presented binaurally by replacing the single tube from the left ear insert phone with a bifurcated tube. The 40-Hz ASSR potentials were evoked by the following two kinds of auditory stimuli with the intensity levels at 70 dBHL. The continuous sine wave tones with a carrier frequency (CF) of 1000 Hz, which have:

(i) 100% depth of amplitude modulation (AM) at 40 Hz: The tone volume sinusoidally changes between 0 and 70 dB at the rate of 40 Hz.
(ii) 100% depth of AM plus 10% width of frequency modulation (FM) at 46 Hz: A sinusoidal change of the tone volume between 0 and 70 dB and a fluctuation of the CF tone between 900 and 1100 Hz occur at the rate of 46 Hz.

The tone (i) is one of the basic stimuli that have been used, though as intermittent tones for ASSR research in schizophrenia^27,37^. The tone (ii) is the stimulus preset in Audera for testing ASSR on awake condition. The tone (ii) is slightly modified from the tone (i) to increase the ASSR potentials by adding FM to AM^38,39^. The ASSRs evoked by tone (i) and tone (ii) were defined in this study as the basic 40-Hz ASSR and the modified 46-Hz ASSR, respectively. The EEG activity to measure the ASSR was obtained from the surface electrode (Neuroline 720 Ambu, DenMark) placed on the forehead around the middle point between the Fz and Fpz of the International 10–20 system. The surface electrodes for the reference and the ground were placed on the left earlobe and the low forehead around Fpz, respectively. The electrode impedances were <5 kOhms.

### ASSR analysis

**Figure 4:**
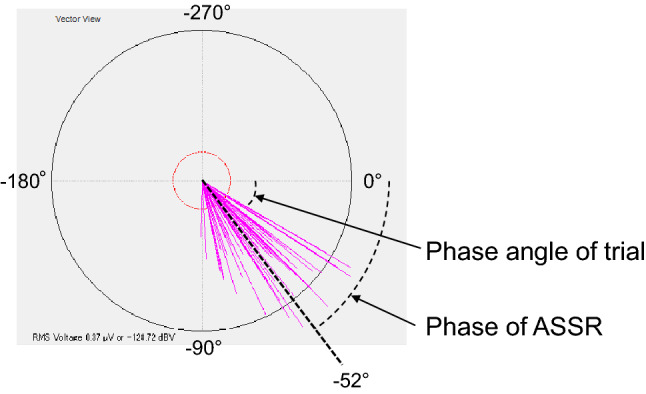
A healthy subject’s result of the basic 40-Hz ASSR in the evoked response audiometry (ERA) system. Each pink vector in the diagram displays the phase angle and the response amplitude of each trial. The phase angle (°) of each vector represents the time-lag from the periodic tone stimuli to the neural response of each trial at the modulation frequency of 40 Hz. The mean of the phase angles of 64 trials was provided as the phase of ASSR in the ERA system (the illustrated straight dotted line, − 52° in this case). The length of each vector represents the response amplitude of each trial. The red inner and the black outer circles represent the lines of 0.1 μV and 0.5 μV, respectively.

The analysis of ASSR was automatically performed by a statistical algorithm adopted in the Audera system. The continuous EEG sampling during the tone stimulation, analog filtered at 0.2–10,000 Hz, was serially segmented into epochs that were overlaid as a trial. The trial was subsequently applied to a fast Fourier transform for calculating the phase angle and the response amplitude in the frequency domain corresponding to the modulation frequency (i.e., 40 and 46 Hz for the basic 40-Hz ASSR and the modified 46-Hz ASSR, respectively). These outcomes of trial were reported as a vector as represented in Fig. 4. The default setting of Audera “terminate test at barriers” automatically terminates the measurement at the time the trials of ASSR statistically reached to a phase-locked response. However, to focus on the evaluation of phase angle in this study, we selected to continue the measurement up to the maximum period, 64 trials, by skipping the judgment of the phase-locked response. Since each trial consists of 10 overlays of EEG epochs in the ASSR analysis, this setting with 64 trials, which takes only 98 s to perform, resulted in as many as 640 overlays of epochs. The phase angles of 64 trials were automatically averaged in an angular way and reported as Phase for the ASSR in the Audera system.

### Statistics

Watson–Williams test was used for circular statistics to examine the phase difference between the two groups as described previously^22^. A significance level of *p* = 0.05 was used for this group analysis between patients with schizophrenia and case-matched healthy subjects. The correlation of phase with clinical variables was examined using Circular-Linear correlation analysis. Because 10 different clinical variables listed in Table 1 were examined, the threshold for the significance of p-values was set at 0.005 (0.05/10) by the Bonferroni correction when analyzing these clinical variables. These circular statistics were conducted using Oriana 4 (Kovach Computing Services, UK) software.

## Data Availability

The data supporting the findings of this study are available from the corresponding author upon reasonable request.
